# Dynamic pathway linking Pakistan flooding to East Asian heatwaves

**DOI:** 10.1126/sciadv.adk9250

**Published:** 2024-04-24

**Authors:** Zheng-Hang Fu, Wen Zhou, Shang-Ping Xie, Ruhua Zhang, Xudong Wang

**Affiliations:** ^1^ Key Laboratory of Polar Atmosphere-ocean-ice System for Weather and Climate, Ministry of Education, Department of Atmospheric and Oceanic Sciences & Institute of Atmospheric Sciences, Fudan University, Shanghai, China.; ^2^ Key Laboratory for Polar Science of the MNR, Polar Research Institute of China, Shanghai, China.; ^3^ Scripps Institution of Oceanography, University of California San Diego, La Jolla, CA, USA.

## Abstract

In July to August 2022, Pakistan suffered historic flooding while record-breaking heatwaves swept southern China, causing severe socioeconomic impacts. Similar extreme events have frequently coincided between two regions during the past 44 years, but the underlying mechanisms remain unclear. Using observations and a suite of model experiments, here, we show that the upper-tropospheric divergent wind induced by convective heating over Pakistan excites a barotropic anomalous anticyclone over eastern China, which further leads to persistent heatwaves. Atmospheric model ensemble simulation indicates that this dynamic pathway linking Pakistan flooding and East Asian heatwaves is intrinsic to the climate system, largely independent of global sea surface temperature forcing. This dynamic connection is most active during July to August when convective variability is large over Pakistan and the associated divergent flow excites barotropic Rossby waves that propagate eastward along the upper troposphere westerly waveguide. This robust waveguide and the time delay offer hopes for improved subseasonal prediction of extreme events in East Asia.

## Teaser

Pakistan flooding can cause East Asian heatwaves through an upper-level waveguide along the Asian westerly jet.

## INTRODUCTION

**Fig. 1.**
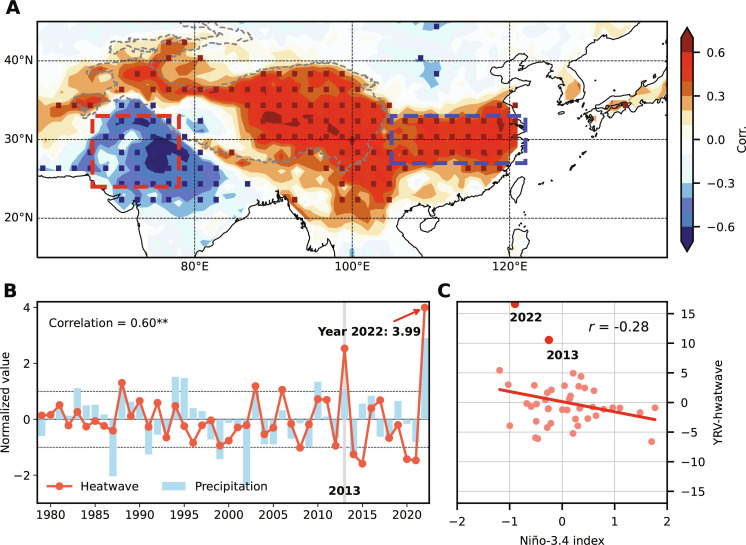
Observed relation between Pakistan rainfall and East Asian heatwave days during July to August. (**A**) Correlations between July through August averaged PNWI (red dashed rectangle; 67°E-79°E and 24°N-33°N) rainfall and gridded heatwave days (shading) over East Asia during 1979–2022. (**B**) Standardized time series of year-to-year July through August averaged rainfall over PNWI (blue bar) and heatwaves (red line) over the Yangtze River valley (YRV; blue dashed rectangle; 105°E-122°E and 26°N-33°N), with one SD dashed. (**C**) Scatter plot for the Niño-3.4 index (K) and YRV heatwave days (day year^−1^) during July to August for 1979–2022. In (A), dotted areas denote that the correlations are 95% significant, and gray dashed curves represent the Tibetan Plateau (TP). Ending with two asterisks in (B) indicates that the correlation is 99% significant.

The Asian summer monsoon system affects the most densely populated regions around the world. Extreme events such as flooding (*1*), heatwaves (*2*), and tropical cyclones (*3*) in these regions could result in a large number of casualties and property losses. During July to August 2022, the worst floods, together with glacial lake bursting, ravaged Pakistan, displacing over 32 million people and causing more than 1200 deaths (*4*, *5*). When Pakistan was under sea-like floodwaters, many regions of China, especially the Yangtze River valley (YRV), suffered the longest-running and most intense heatwaves since 1961, further leading to historic drought in southern China (*6*). These heatwave-induced compound extreme events highlight the importance of identifying physical drivers and improving the prediction of such extreme events in changing climate. The co-occurrence of Pakistan flooding and East Asian heatwaves is not uncommon in historical observations. For instance, major flooding in Pakistan (*7*) and severe heatwaves in southern China (*8*) were also simultaneously reported during July to August 2010. On the interannual timescale, the correlation between Pakistan–northwestern Indian (PNWI) precipitation and YRV heatwaves reaches 0.60 during July to August, significant at the 99% confidence level (Fig. 1). This robust in-phase relationship between the two extreme events has been noted in several studies (*9*, *10*), but the underlying mechanisms remain to be determined.

The El Niño–Southern Oscillation (ENSO) is a coupled atmosphere-ocean phenomenon with global impacts (*11*–*13*). During summers when El Niño develops, the weakened Walker circulation suppresses the South Asian summer monsoon rainfall (*14*, *15*), and the anomalous anticyclone (AAC) over the western North Pacific (WNP) affects the climate in East Asia by inducing the westward extension of the subtropical high (*16*). During post–El Niño summers, the delayed response of Indian Ocean sea surface temperature (SST) to ENSO anchors the WNP AAC through a warm tropospheric kelvin wave (*17*, *18*). As a key driver of climate in the Asian summer monsoon, Jeong *et al.* (*19*) suggested that the strong negative Indian Ocean dipole event driven by the recent triple-dip La Niña could contribute to the Pakistan flooding and southern China drought in the summer of 2022. However, the SST-forced pattern in (*19*) underestimates the precipitation anomalies of the two extreme events, and the proposed correlation between ENSO and East Asian heatwaves is only marginal (Fig. 1C). During midsummer 2013, extreme heatwaves hit southern China in duration second only to 2022, while only small SST anomalies emerged in the equatorial Pacific from 2012 to 2014 (Fig. 1, B and C). On the other hand, PNWI at the same time experienced above-normal rainfall, reaching +1.07 SDs (Fig. 1B). Therefore, the SST forcing could not explain the whole story.

Atmospheric internal variability is a key modulator of extreme events in Asian summer monsoon regions (*20*–*22*) but limits the seasonal predictability due to the stochastic nature of the atmosphere. The circumglobal teleconnection (CGT) pattern (*23*, *24*), also known as the Silk Road pattern (SRP) (*25*), is an important atmospheric internal mode that affects PNWI flooding (*26*) as well as East Asian heatwaves (*27*) through stationary Rossby waves along midlatitudes. The possible linkages between Indian summer rainfall and CGT have been reported in (*23*), and a subsequent modeling study indicated that Indian summer rainfall could reinforce the CGT in downstream regions (*28*). In studying the concurrent extreme events of 2022, Tang *et al.* (*29*) attributed the record-breaking heatwaves over the YRV to Pakistan flooding–reinforced CGT and La Niña–enhanced WNP subtropical high. However, it should be noted that tropical SST forcing, such as ENSO and Pacific meridional mode, also simultaneously modulates both the Pakistan rainfall (*14*, *30*) and CGT pattern (*24*, *30*, *31*). Therefore, the relative roles of SST forcing and atmospheric internal variability remain to be quantified to resolve contradictions among recent studies (*19*, *29*, *32*, *33*). While previous studies emphasize role of the upstream impacts from CGT (*9*, *23*, *24*, *29*), recent evidence suggests that low-pressure systems known as monsoon depressions originating from the tropics seem to be more important for Indian summer rainfall extremes (*34*), such as the southern Pakistan mega-flooding in midsummer 2022 (*35*). This challenges the idea that CGT acts as the primary initiator of simultaneous Pakistan flooding and East Asian heatwaves. Moreover, although recent studies propose a potential connection between Pakistan flooding and East Asian heatwaves, the specific feedback mechanisms underlying this connection have not been systematically explored so far (*32*). Several important questions arise: How do SST forcing, such as ENSO, and internal variability contribute to the strong co-occurring tendency between Pakistan flooding and East Asian heatwaves? What is the dynamic pathway through which Pakistan rainfall exerts influence on East Asian climate, especially long-lasting heatwaves?

The present study investigates these questions based on a wide range of observations and global SST-forced atmospheric model simulations. We show that the co-occurrence of Pakistan flooding and East Asian heatwaves is an internal mode intrinsic to the Asian summer monsoon system, largely independent of ENSO. Experiments with the linear baroclinic model (LBM) confirm the pathway linking extreme events in these two regions through interactions between convection-induced upper-tropospheric divergent flow and the subtropical westerly jet. This dynamic pathway is sensitive to the heating location and most active in July to August. We show that convective variability over Pakistan is optimal to excite barotropic perturbations over China. This upper-tropospheric pathway’s tight correlation with surface conditions proves its value in predicting extreme events at a subseasonal timescale.

## RESULTS

### Covariability of Pakistan flooding and East Asian heatwaves

**Fig. 2.**
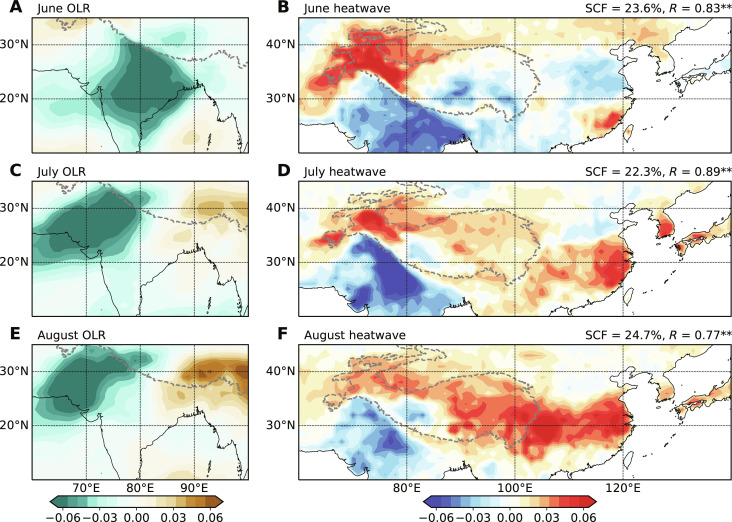
Monthly evolution of coupled pattern between South Asian convection and East Asian heatwave days for 1979–2022. The left panels show the patterns of outgoing longwave radiation (OLR; shading) over South Asia (60°E-100°E, 10°N-35°N) that accompany the first mode in maximum covariance analysis (MCA) for (**A**) June, (**C**) July, and (**E**) August. The right panels show the associated patterns of heatwaves (shading) over East Asia (60°E-140°E, 20°N-45°N) for (**B**) June, (**D**) July, and (**F**) August. In (A) to (F), gray dashed curves represent the TP. In (B), (D), and (F), the squared covariance fraction (SCF) indicates the portion of covariance explained by the leading mode, and *R* denotes the correlation between the time series of the OLR and heatwave mode from June to August. Endings with two asterisks indicate that *R* is 99% significant.

**Fig. 3.**
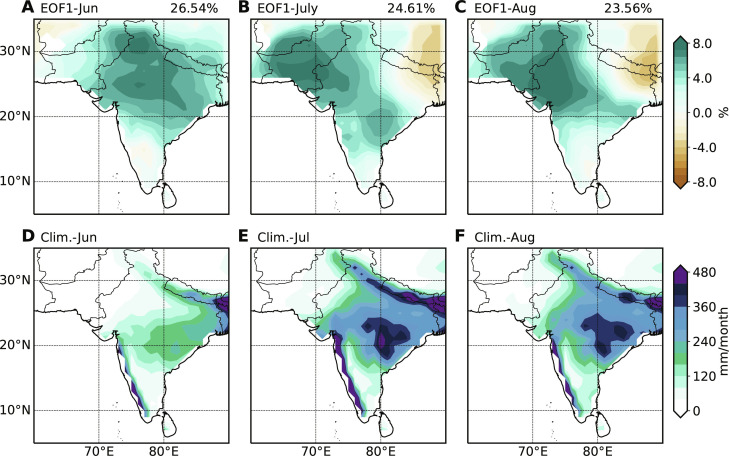
Variability and climatology of Indian summer monsoon rainfall. (**A** to **C**) Dominant pattern from monthly empirical orthogonal function (EOF) decomposition for standardized Indian summer monsoon rainfall during 1901–2022. (**D** to **F**) Monthly mean rainfall (shading; mm month^−1^) over the Indian summer monsoon region during 1901–2022. Explained variance of the leading mode is denoted at the top of (A) to (C).

As a first step, we consider the spatially coherent structure of outgoing longwave radiation (OLR) in South Asia and heatwaves in East Asia using maximum covariance analysis (MCA) from June to August 1979–2022 based on the fifth-generation atmospheric reanalysis from the European Centre for Medium-Range Weather Forecasts (ERA5; Fig. 2). The OLR patterns exhibit noticeable monthly evolutions, with large variability moving from central India in June to PNWI in July to August. The leading empirical orthogonal function (EOF) modes obtained for precipitation over the Indian summer monsoon region yield almost the same pattern as extracted in MCA (Fig. 3, A to C), suggesting that the coupled mode from MCA captures the interannual variability in Indian summer monsoon rainfall (*36*). Furthermore, the correlations between the OLR temporal coefficients and the all-Indian rainfall index (*37*) from June to August reach 0.81, 0.66, and 0.76, respectively. This indicates that the coupled patterns could also serve as good indicators of overall convection intensity for the Indian subcontinent (also see Fig. 3, D to F). In light of these, the subsequent analysis will concentrate on the covariability between PNWI convection and East Asian heatwaves. From July to August, the enhanced convection over PNWI is coupled with increased heatwaves in the YRV and much of the Tibetan Plateau (TP). However, the leading mode from MCA in June does not feature a simultaneously enhanced heatwave over the YRV and its vicinity (Fig. 2B) for the reasons to be discussed below.

**Fig. 4.**
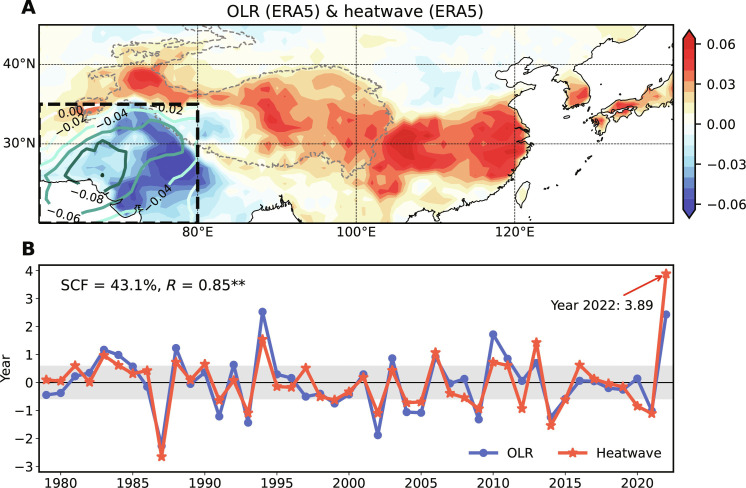
Coupled pattern between July through August averaged northwestern South Asian convection and East Asian heatwaves. (**A**) Results for July through August averaged northwestern South Asian (60°E-80°E, 20°N-35°N) OLR (contours) and East Asian heatwaves (shading) that accompany the first mode of MCA for 1979–2022. (**B**) Time series of the OLR (blue line) and heatwave (red line) patterns, with ±0.6 SD shaded. In (A), gray dashed curves represent the TP. The SCF and temporal correlation (R) are denoted at the top of (B). Endings with two asterisks indicate that the correlation is 99% significant in (B).

In the rest of the paper, we focus on covariability in July through August averages between PNWI OLR and East Asian heatwaves (Fig. 4). The OLR anomaly field reveals a southwest-northeast slanted feature with a center over southern Pakistan, which is accompanied by increased heatwave occurrences from the TP (except the southwestern portion) through the YRV to western Japan (Fig. 4A). The coupled pattern shows a northwestward expansion of the Indian summer monsoon and a local enhancement of summertime heatwaves along a wide swath of East Asia. More specifically, the Pakistan mega-flooding and YRV record-breaking heatwaves in midsummer 2022 are well captured in our MCA mode, with OLR and heatwave anomalies reaching 2.43 and 3.89 SDs (Fig. 4B). Other similar extreme events are identified in 2010 and 2013 (*7*, *8*, *38*, *39*).

In addition, we repeat the MCA using OLR from National Oceanic and Atmospheric Administration (NOAA) and the heatwave data calculated from Berkeley Earth Surface Temperature (BEST). The leading pattern is also characterized by enhanced convection over PNWI and increased heatwaves from the eastern TP to western Japan (fig. S1A). The minor distinctions among spatial patterns, such as the different heatwave patterns over the western TP and Sichuan basin, are mainly due to different investigation periods (Fig. 4A and fig. S1A). Correlations of the time series between the two MCA patterns reach 0.93 for OLR and 0.94 for heatwaves (fig. S1B). These near-perfect correlations suggest that our coupled mode of Pakistan flooding and East Asian heatwaves is robust on the interannual timescale and is insensitive to the dataset selection.

### Atmospheric dynamic processes

To investigate the atmospheric dynamic processes connecting Pakistan flooding and East Asian heatwaves, we perform a composite analysis concerning the mode coefficients (Fig. 4B). Our positive group includes the years in which both the OLR and heatwave mode exceed +0.6 SD, and negative group contains the years in which these two indexes are below −0.6 SD (table S1). We choose ±0.6 SD as the threshold to maintain an appropriate sample size, and the following composite results remain almost unchanged if the threshold is gradually altered from ±0.4 to ±0.8.

**Fig. 5.**
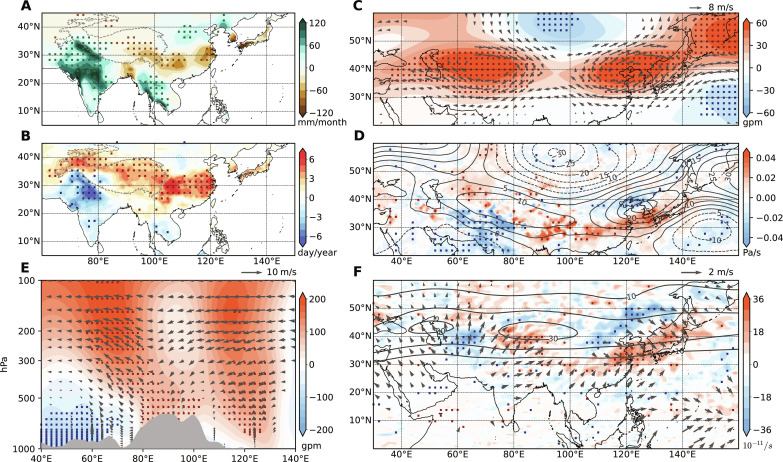
Atmospheric circulations associated with the coupled mode of Pakistan flooding and East Asian heatwaves. Composite pattern differences of horizontal fields for (**A**) rainfall (shading; mm month^−1^), (**B**) heatwaves (shading; day year^−1^), (**C**) 200 hPa winds (vectors; m s^−1^) and geopotential height (shading; gpm), and (**D**) 500 hPa vertical velocity (shading; Pa s^−1^) and geopotential height (contours at 5 gpm interval; gpm). (**E**) Composite vertical differences of meridional-integrated zonal wind (m s^−1^) and vertical velocity (ω, multiplied by 100; vectors; Pa s^−1^) and geopotential height (shading; gpm) averaged between 26°N and 33°N. (**F**) Composite differences in the Rossby wave source (RWS; shading; 10^−11^ s^−1^) and divergent wind (vectors; m s^−1^) at 200 hPa. In (A) to (F), the dotted area indicates that the difference is significant at the 95% confidence level. The TP is denoted by gray dashed curves in (A) and (B). In (C), (E), and (F), we display the vectors only where differences are 95% significant. The climatology of the mean zonal wind ([10, 20, 30] m s^−1^ gray contours) at 200 hPa during July to August is overlaid in (F).

The composite analysis reaffirms the connection between the enhanced precipitation over PNWI and intensified heatwaves over YRV and its vicinity (Fig. 5, A and B). The rainfall pattern over the Indian subcontinent shows some orographic effects. Middle to low-level wind anomalies transport abundant moisture from the Bay of Bengal and Arabian Sea to northwestern South Asia (fig. S2). Wind confluence occurs over PNWI due to the mechanical effects of the Sulaiman Mountains, Kirthar Mountains, and Himalayas. Therefore, large rainfall anomalies are anchored on the windward slope of high mountains or in the strengthened monsoon trough due to orographic convergence (Fig. 5A and fig. S2). In addition, there is a meridional dipole pattern of precipitation variation, with increased (decreased) rainfall over the northern (southern) eastern China (Fig. 5A). On the south flank of the East Asian AAC, intensified heatwaves are associated with anomalous downward motion (Fig. 5, C and D). Because of the effect of soil humidity and cloud fraction, enhanced rainfall suppresses heatwaves over PNWI (Fig. 5B). At 200 hPa, there are two AACs over the western and eastern of the TP, respectively, with distinctive dynamic structures. The AAC over the western TP exhibits a baroclinic structure weak in geopotential height at 500 hPa, while an equivalent barotropic AAC occurs over the eastern TP (Fig. 5, C and D). Especially, the atmospheric circulation anomalies are both significant in the upper and lower troposphere over northwestern South Asia (Fig. 5B and fig. S2B), which corresponds to a strong “convection-circulation” feedback mechanism (*40*). However, the upper-level AAC seems to be the dominant feature responsible for the intensified heatwaves over East Asia (Fig. 5C and fig. S2B), with a weak high-pressure system and insignificant wind anomalies in low-level circulations.

Vertical transect averaged between 26°N and 33°N along Asian regions further shows the effect of convection over PNWI on circulations, with negative geopotential height near the surface and released latent heating thickening the atmosphere from 500 hPa to the upper troposphere (Fig. 5E). Especially, positive geopotential height anomalies dominate through the troposphere over the western TP. Downward vertical velocity prevails to the east of 90°E over East Asia, with increased geopotential height from the surface to the upper troposphere, indicative of an equivalent barotropic Rossby wave (Fig. 5E). This downward motion possibly arises from cold horizontal advection by easterly anomalies due to strong zonal temperature gradient near TP and the suppressed local convection (*40*, *41*). Furthermore, the convectively lofted air masses in PNWI induce strong divergent flow over the upper troposphere (Fig. 5F), which effectively produces a negative Rossby wave source (RWS) near the subtropical westerly jet over northwestern Pakistan. The subtropical westerly jet here acts as a waveguide, enabling negative RWS to excite a stationary barotropic Rossby wave train downstream. Cyclonic circulation over Mongolia and the AAC over eastern China could be excited in this way. In addition, the positive RWS over the YRV is possibly due to the strong convergence induced by locally suppressed convection, and the associated negative RWS to the north could further strengthen the AACs over eastern China and the downstream regions.

Up to now, we have demonstrated the large-scale atmospheric dynamics associated with the leading mode in MCA. Possible physical connections between Pakistan convection and East Asian heatwaves are determined based on observations. Note that the in-phase correlation could also come from a common external forcing, which modulates Pakistan rainfall and East Asian heatwaves at the same time. In the next section, we analyze an ensemble of 10-member Atmospheric Model Intercomparison Project (AMIP) (*42*) simulations with the Community Atmosphere Model version 6 (CAM6) of the Community Earth System Model version 2 (CESM2) (*43*) to assess the relative contributions of global SST forcing and atmospheric internal variability to the coupled mode of Pakistan flooding and East Asian heatwaves.

### Global SST forcing versus atmospheric internal variability

We first assess the relation of PNWI OLR and YRV heatwaves with global SST in observations (fig. S3, A and B). The most prominent SST pattern is the cooling over the eastern tropical Pacific, which led us to focus on the influence from ENSO on the simultaneous extreme events. The lagged correlations between Niño-3.4 with PNWI OLR peak in June to August, suggesting a concurrent ENSO influence (fig. S3C). However, the correlations between ENSO and YRV heatwaves are only marginal. To examine the possible ENSO influence on observed in-phase relations between PNWI rainfall and YRV heatwaves, we conduct correlation tests on four precipitation datasets and two heatwave datasets (see details in Materials and Methods) (Table 1). The raw correlations between PNWI rainfall and YRV heatwaves are robust among all datasets, ranging from 0.47 to 0.62, while the correlations between PNWI rainfall and ENSO are marginally negative. This precludes the possibility that ENSO affects East Asian heatwaves by modulating PNWI convection. The correlations between YRV heatwaves and ENSO increase after removing PNWI rainfall signals (table S2), while correlations between PNWI rainfall and YRV heatwaves slightly increase after regressing out the ENSO influence (Table 1). This implies that the connection between PNWI rainfall and YRV heatwaves is largely independent of ENSO.

**Table 1.** Observed correlations between PNWI precipitation with YRV heatwaves and the ENSO during 1979–2014. We use four precipitation datasets and two heatwave datasets (see Materials and Methods). Besides directly correlating the two series, we perform partial correlations to filter out the possible influence of ENSO (no ENSO). The correlations reaching ±0.33/±0.39 here are at a 95%/99% confidence interval.

PNWI precipitation	YRV heatwaves				ENSO
	ERA5	ERA5 (no ENSO)	BEST	BEST (no ENSO)	
CRU	0.57	0.58	0.47	0.52	−0.35
ERA5	0.58	0.59	0.53	0.58	−0.31
CMAP	0.62	0.62	0.53	0.57	−0.24
GPCP	0.60	0.61	0.50	0.54	−0.30

We use CESM2-CAM6 AMIP simulations to further evaluate the relative role of global SST forcing and atmospheric internal variability. We first evaluate the model performance in reproducing the observed relation among PNWI rainfall, YRV heatwaves, and ENSO (table S3). Note that we use surface temperature in place of heatwave days, which has less uncertainty in current global atmospheric models (*44*). Results indicate that individual members of CESM2-CAM6 could well simulate the observed relation, with correlations falling within the observed ranges (table S3).

**Fig. 6.**
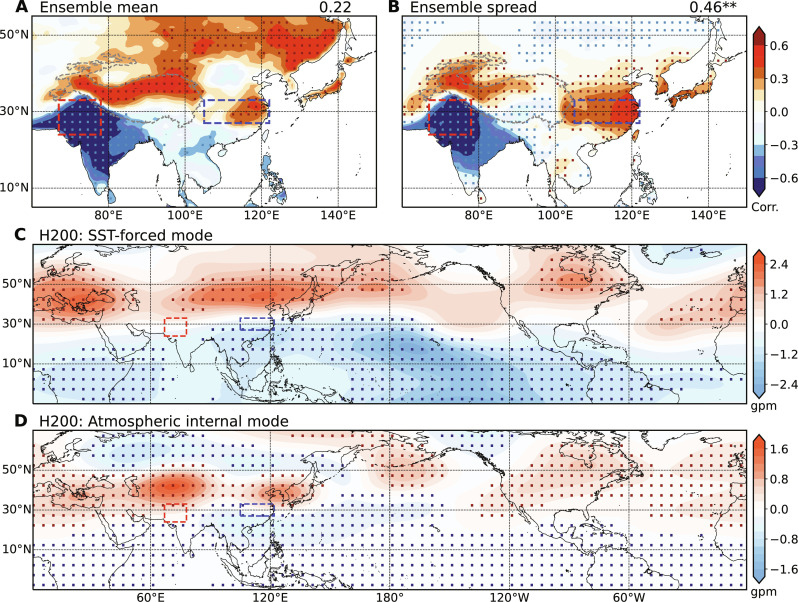
Global SST-forced pattern and atmospheric internal pattern related to Pakistan convection. Correlations between averaged PNWI OLR (multiplied by −1; W m^−2^) and gridded surface temperature (K) over East Asia from July through August for the (**A**) ensemble mean and (**B**) ensemble spread. Geopotential height at 200 hPa (shading; gpm) regressed against the averaged OLR in PNWI (multiplied by −1; W m^−2^) for the (**C**) ensemble mean and (**D**) ensemble spread. The correlations between averaged PNWI OLR and YRV surface temperature are denoted in the upper right of panels (A) and (B), and the correlation ending with two asterisks in (B) exceeds the 99% confidence level. In (A) to (D), dotted regions denote results significant at the 95% confidence level.

Correlation between AMIP ensemble-mean PNWI OLR and YRV temperature is insignificant, indicating the weak influence of SST forcing (Fig. 6A). Correlations are significant in the northern TP and Yangtze Plain, suggestive of the influence of SST-forced PNWI convection. The PNWI OLR-YRV temperature correlation in the AMIP ensemble spread is strongly negative, reaching −0.46 over 1140 years (Fig. 6B). The geopotential height pattern at 200 hPa regressed onto PNWI OLR features a zonally symmetric seesaw pattern in the AMIP mean results, with a uniform cooling in the tropics and comparable warming in the extra-tropics (Fig. 6C). This SST-forced circulation pattern closely resembles the developing ENSO-forced pattern and contributes to the CGT across the Northern Hemisphere (*24*). In contrast, the atmospheric internal mode in the ensemble spread features geopotential anomalies in the extra-tropics, with two prominent upper-level AACs within 30°N-50°N over the Asian summer monsoon region (Fig. 6D). This pattern is reminiscent of the observed MCA mode of Pakistan convection and East Asian heatwaves (Fig. 5C). Although the geopotential height pattern looks similar within 30°N-50°N between the ensemble mean and spread, the precipitation correlation patterns are distinct (fig. S4). The SST-forced pattern is of a global scale while the internal mode is confined mostly within the Asian summer monsoon region. Furthermore, the correlation in each member of AMIP simulations between global SST with PNWI OLR and YRV temperature does not show any prominent patterns (fig. S5), consistent with the findings from the AMIP ensemble mean (fig. S4A). Together, these results indicate that SST forcing plays a minor role in linking Pakistan flooding and East Asian heatwaves.

Consistent with observations, the wave train connecting PNWI convection and East Asian heatwaves disappears in June in model simulations (fig. S6). This is because convective variability is displaced southeast, away from the upper-level westerly waveguide (fig. S7). These results further illustrate that the connection of PNWI convection and East Asian heatwaves is inherent to wave dynamics of the upper-level Asian westerly jet.

### LBM experiments

**Fig. 7.**
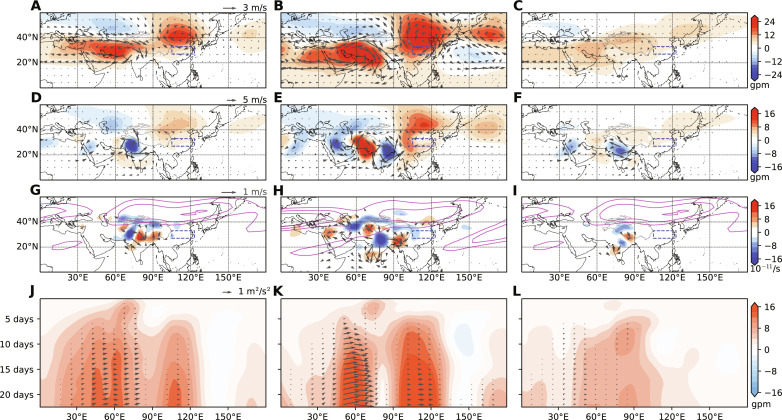
Atmospheric responses to deep heating over Pakistan in LBM experiments. (**A** to **C**) 200 hPa geopotential height (shading; gpm) and horizontal wind fields (vectors; m s^−1^) in exps. 1 to 3, respectively. (**D** to **F**) The same as in (A) to (C) but for 850 hPa. (**G** to **I**) The same as in (A) to (C), but for 200 hPa RWS (shading; 10^−11^ s^−1^) and divergent wind (vectors; m s^−1^). The climatologies of mean zonal wind ([15, 20, 25] m s^−1^ gray contours) at 200 hPa in each experiment are denoted in (G) to (I). (**J** to **L**) Hovmöller plots of 200 hPa wave activity flux (WAF; vectors; m^2^ s^−2^) and geopotential height (shading; gpm) averaged over 30°N-40°N. The abscissa is longitude, and the ordinate is the integration time (day) from day 1 to day 25.

To confirm the dynamic pathway through which Pakistan flooding could contribute to East Asian heatwaves, we performed three experiments with LBM (see Materials and Methods and fig. S8). With convective heating over PNWI, the LBM reproduces the two observed upper-tropospheric AACs (Fig. 7A). The western AAC is of a baroclinic structure, with low-level southwesterlies and convergences over PNWI. This indicates the positive feedback between convective heating and low-level circulations. The eastern AAC over eastern China features a quasi-barotropic stationary Rossby wave structure (Fig. 7, A and D). In detail, the upper-tropospheric divergent flow associated with PNWI convective heating produces a negative RWS near the south flank of the subtropical westerly jet, leading to the downstream AAC response over East Asia (Fig. 7, A, D, and G). In comparison with observations, the negative RWS over the YRV is not found in the LBM results (Figs. 5F and 7G). This indicates that the AAC over eastern China could be directly forced by PNWI convection and then acts to suppress local convection, resulting in upper-tropospheric convergence and RWS over northeast China near the westerly jet. The RWS further reinforces the eastern AAC through “convection-circulation” feedback (*45*).

The second experiment tests the sensitivity to the background flow. With a June basic flow, the atmospheric responses are more prominent than those in exp. 1 (Fig. 7, A, B, D, and E). The larger anomalies are due to a more southerly location of the June climatological westerly jet, which acts as an effective waveguide for disturbances from Pakistan. These results suggest that Pakistan convection could force a strong Rossby wave response on a June basic state but convective variability is weak over PNWI and this dynamic pathway is not well developed as a result.

While Indian summer monsoon convection has been suggested to influence the circulations in downstream regions [e.g., in (*23*, *24*, *28*)], results of our third experiment (with convective heating over central India) do not support this conclusion. Typically, rainfall in central India reaches 250 to 500 mm month^−1^ during July to August, far more than in Pakistan (Fig. 3, E and F). Diabatic heating in central India, however, does not yield strong downstream stationary wave responses along the upper-level jet of July to August (Fig. 7C). RWS is dominated by the vortex stretching term (see Materials and Methods), which is largely proportional to the planetary vorticity (*f*). Hence, RWS is weak for a convective heating over lower-latitude central India and does not extend north far enough to reach the westerly jet in July to August, producing only weak atmospheric anomalies in LBM exp. 3 (Fig. 7, C, F, and I). These results indicate that although rainfall is large in midsummer over central India, it does not effectively excite the Rossby wave response.

We then use Hovmöller diagrams to track the evolution of the response to diabatic heating (Fig. 7, J to L). The geopotential height anomalies extend to the west of heating in the first several days, indicative of a baroclinic Rossby response (*46*, *47*). Wave activity flux (WAF) shows an eastward energy propagation of Rossby waves. The eastern AAC in exp. 1 and exp. 2 gradually develops over 7 days after the heating is imposed, anchored near 105°E-115°E. In the end, the atmospheric anomalies are mainly confined to Asian summer monsoon regions, with a weak response east of 130°E. The delayed response in East Asia is important for subseasonal prediction. In addition, the absence of Rossby wave response in exp. 3 suggests that the heating location over central India is not optimal for impacts on East Asia through the westerly waveguide.

## DISCUSSION

**Fig. 8.**
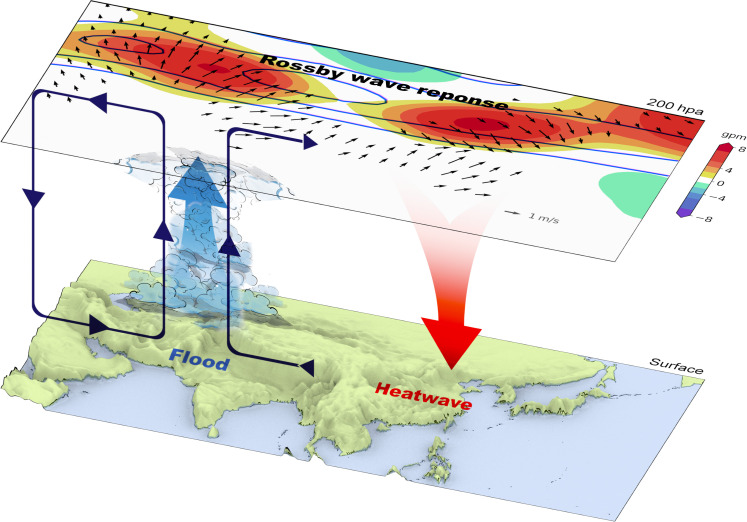
Schematic of the dynamic pathway linking Pakistan flooding to East Asian heatwaves. The 200 hPa geopotential height (shading; gpm) and divergent wind (vectors; m s^−1^) come from Fig. 5 (C and F, respectively). The climatology of the mean zonal wind ([10, 20, 30] m s^−1^ contours) at 200 hPa during July to August is also overlaid. The terrain shown in the lower panel comes from the Global Multi-resolution Terrain Elevation Data 2010 (GMTED2010) (*66*).

We have used observations, AMIP simulations, and LBM experiments to show that the connection of Pakistan flooding and East Asian heatwaves is dynamically intrinsic to the Asian summer monsoon system, largely independent of ENSO forcing. Figure 8 shows a schematic of the atmospheric dynamic pathway. Deep convection over PNWI induces a baroclinic AAC to the west much as the direct Matsuno-Gill response. In addition, the divergent wind in the upper troposphere produces barotropic Rossby waves with eastward energy propagation along the subtropical westerly jet. Hence, negative RWS produced by convective heating leads to the subsequent AAC over eastern China, which is favorable for long-lasting heatwaves and suppresses the local convection. The suppressed convection over the YRV further induces a pair of RWSs, which in turn amplify the circulation anomalies over East Asia. This positive feedback between convection and circulations is crucial to maintain the dynamic pathway connecting Pakistan flooding and East Asian heatwaves. Climatologically, convective precipitation spreads northwestward over India from June to July (Fig. 3, D and E), and the interannual variability is large on the northwestern edge of monsoon convection (Fig. 3, B and C). During July to August, the ratio of the SD of precipitation to the mean is 15 to 20% at the center of monsoon convection but increases to 50 to 200% over PNWI. Although central India is where the strongest convective activities take place, because of dynamic limitations and weak convective variability, these activities fail to effectively excite circulation responses, and Pakistan flooding turns out to be an effective modulator of summer climate in East Asia. Our study highlights that the impact of convection over the Indian subcontinent on downstream regions from July to August is not as strong as previously thought.

AMIP simulations confirm that the coupled mode of Pakistan flooding and East Asian heatwaves is largely controlled by atmospheric internal variability, independent of ENSO influence. Although the upper-tropospheric circulations have a lot in common with the global-scale CGT/SRP, our coupled pattern–related atmospheric responses are mainly confined to the Asian summer monsoon system, which is directly modulated by strong diabatic forcing from the sweet spot, Pakistan (fig. S4B).

In the classical view, heatwaves over East Asia are directly modulated by the WNP subtropical high [e.g., in (*8*, *29*, *39*, *48*)], although their correlation is moderate on interannual timescale [only 0.44 for the YRV; figure 2D in (*29*)]. What complicates the matter even more is that the relationship between ENSO and the WNP AAC is not stable through time (*49*, *50*), and the associated rainfall variability is confined to the eastern flank of the TP (*51*). Hence, the driving factors of heatwaves over East Asia are difficult to ascertain when we focus on low-level circulations. We identified an upper-tropospheric pathway linking Pakistan flooding to an AAC over East Asia, which generally occurs at preferred locations due to the internal dynamics of the basic-state flow (*24*, *52*). In addition, the upper-tropospheric zonal wind anomalies related to this AAC contribute to mid-level vertical motion anomalies, further influencing the surface climate conditions (*9*). This relationship is very robust, with correlations reaching as high as ~0.9 (fig. S9, A and B). These insights are valuable for improving subseasonal prediction of extreme events in East Asia through a westerly waveguide in the upper troposphere.

It is worth noting that the amplified warming induced by convection heating over Pakistan can lead to heatwaves over the western TP (Figs. 1A and 5B), where large amounts of snow remain in summer (*53*). Therefore, the melting of snow in these regions can reduce the surface albedo, further reinforcing the local heatwaves and upper-level AAC. On the one hand, the strengthened AAC could enhance Pakistan convection through increased easterly vertical wind shear (*54*). On the other hand, snow melting and glacial lake breaching could directly contribute to the Pakistan floods as reported in 2022 (*4*). This “monsoonal heating-glacial melting” positive feedback mechanism deserves further investigations.

In a warming climate, ENSO-unrelated summer variability in the Indo-Western Pacific is likely to intensify (*55*), highlighting the increasing importance of internal variability. Furthermore, Pakistan rainfall extremes could be worsened by anthropogenic warming through enhanced cross-equatorial moisture transport over the Arabian Sea (*35*). A moderate increase in the occurrence or magnitude of simultaneous extreme events over populous South and East Asia as in 2022 could exacerbate socioeconomic consequences.

## MATERIALS AND METHODS

### Heatwave definition

A heatwave event is defined as when the daily maximum temperature (*T*_max_) reaches the threshold temperature for at least three consecutive days. Here, the threshold temperature is determined by the 90th percentile of daily *T*_max_ based on a 15-day window surrounding each calendar day during 1981–2010 (*56*, *57*). Such a daily-based percentile definition considers the subseasonal variation of the climatology and enables inter-regional comparison with its homogenous distribution. We calculate the total number of days of heatwave events every year from June to August.

### Observational datasets

For heatwave calculation, we use hourly *T*_max_ at 2 m from ERA5 and process it to daily *T*_max_ during 1979–2022 (*58*). Daily *T*_max_ from the BEST dataset available from 1979 to 2019 is also used for validation (*59*). For monthly averaged temperature, zonal and meridional wind fields, vertical velocity, and geopotential height, we use the ERA5 reanalysis during 1979–2022 with 37 vertical pressure levels from 1000 to 1 hPa (*58*). Monthly averaged OLR is also obtained from ERA5 during 1979–2022 (*58*), and monthly OLR data from NOAA are used for data validation (*60*). Monthly land precipitation data are from the Climate Research Unit, version TS 4.07 (CRU TS4.07), which are station-based data from 1901 to 2022 (*61*). Another three monthly precipitation datasets are used for comparison during 1979–2014: (i) ERA5 total precipitation (*58*), (ii) CPC Merged Analysis of Precipitation (CMAP) (*62*), and (iii) Global Precipitation Climatology Project (GPCP) (*63*). We also use the Extended Reconstructed Sea Surface Temperature version 5 (ERSST.v5) from NOAA during 1901–2022 (*64*). The Niño-3.4 index is defined as a 5-month running mean SST within 5°S-5°N and 170°W-120°W (*65*). The terrain data come from the Global Multi-resolution Terrain Elevation Data 2010 (GMTED2010) (*66*).

### Leading mode extraction

We perform MCA (*67*) to extract the leading coupled mode of northwestern South Asian (60°E-80°E, 20°N-35°N) convection and East Asian (60°E-140°E, 20°N-45°N) heatwaves (Figs. 2 and 4). The results remain almost unchanged with minor jitters in the selected domain. To investigate whether this coupled mode is tied to the fundamental variability of local climate, we also conduct an EOF (*68*) decomposition to extract the dominant mode of precipitation over the South Asian summer monsoon region (60°E-90°E, 5°N-35°N) from June to August (Fig. 3, A to C). We perform EOF analysis on the standardized Indian summer monsoon rainfall field. Hence, pattern values can be viewed as regression coefficients of standardized rainfall on the temporal coefficients (*36*). On the basis of the Monte Carlo technique (*69*) and North test (*70*), the leading modes obtained from the MCA and EOF methods in this study are both well separated from the second mode at a 95% confidence interval.

### Rossby wave diagnosis

A tropical convection anomaly can induce anomalous vertical motion, and the associated upper-level perturbations can produce an anomalous vorticity source, denoted by the RWS. Following Sardeshmukh and Hoskins (*71*), RWS is given as

$$\text{RWS}=-\eta D-{\bar{u}}_{\chi }\cdot \nabla \eta$$ (1)

where η is the absolute vorticity, *D* is the magnitude of divergence, and ${\bar{u}}_{\chi }$ is the divergent component of wind. The vortex stretching term (−η*D*) represents the vorticity generation by divergence, and the absolute vorticity advection by divergence flow ( $-{\bar{u}}_{\chi }\cdot \nabla \eta$ ) is provided by regions with a strong vorticity gradient (e.g., near the subtropical westerly jet) (*72*). Here, we calculate the sum of the two terms but note that the vortex stretching term dominates the RWS.

WAF is independent of the wave phase and parallel to the local group velocity of stationary Rossby waves for slowly varying basic flows (*73*), suitable for investigating Rossby wave propagation in the upper troposphere. The horizontal WAF is given as

$$\text{WAF}=\frac{1}{2|\bar{U}|}\left[\begin{array}{c} \bar{u}\left({\psi \prime }_{x}^{2}-\psi \prime {\psi \prime }_{\mathrm{xx}}\right)+\bar{v}({\psi \prime }_{x}{\psi \prime }_{y}-\psi \prime {\psi \prime }_{\mathrm{xy}}^{2})\\ \bar{u}\left({\psi \prime }_{x}{\psi \prime }_{y}-\psi \prime {\psi \prime }_{\mathrm{xy}}\right)+\bar{v}\left({\psi \prime }_{y}^{2}-\psi \prime {\psi \prime }_{\mathrm{yy}}^{2}\right) \end{array}\right]$$ (2)

where $\bar{U}=\left(\bar{u},\bar{v}\right)$ is the climatological horizontal wind field, and ψ′ = *gZ_a_*/*f* is the perturbation stream function derived from the gravitational acceleration (*g* = 9.8 m s^−2^), geopotential height anomaly (*Z_a_*), and *f* through the use of quasi-geostrophic approximation. Subscript *x* represents the longitudinal derivative (*∂*/∂λ), and *y* denotes the latitudinal derivative (*∂*/∂ϕ).

In this study, we analyze the RWS and WAF at 200 hPa to construct the dynamic pathway linking Pakistan flooding to East Asian heatwaves. Calculations of the RWS and WAF are conducted by the Python package “windpharms” (*74*).

### AMIP simulation

To evaluate the relative importance of global SST forcing and atmospheric internal variability, we use a 10-member ensemble of CAM6-AMIP (*42*) simulations in CESM2 (*43*). The CAM6 model is run at 0.95° × 1.25° horizontal resolution with 70 vertical levels, forced with historical SST from the ERSST.v5 dataset. As the 10 members share the same external forcing, the ensemble mean represents the prescribed global SST-forced variability. Limited by the AMIP output, we use a 36-year period to assess the effects of SST forcing during 1979–2014, in comparison with observations. We calculate the ensemble spread by subtracting the ensemble mean from each member. This will capture the atmospheric internal variability due to the randomized initial conditions of each member run. We concatenate all 10-member runs from 1901 to 2014 in a sequence to investigate internal variability, and in this way, we obtain a 1140-year series.

### LBM experiments

To verify the impact of PNWI convection on East Asian heatwaves, we perform three LBM experiments based on observed climatology of monthly mean fields (ERA5) using a time integration method for 30 days (*75*). Unlike other complicated atmospheric models, LBM only includes linear processes and thus can more accurately produce the circulation anomalies in response to diabatic heating. We use a version with T42 spherical harmonic spatial resolution and 20 vertical sigma levels (T42L20). The vertical profile of heating follows a gamma function with a maximum heating of about 6 to 7 K day^−1^ at 450 hPa, typical of deep convection (*40*). In this work, the response on day 30 is taken as the steady solution.

Exp. 1 investigates the direct response of the circulation to convective heating over Pakistan during July to August. Exp. 2 and exp. 3 are also performed to explore the spatial and temporal sensitivity of the effectiveness of latent heating in South Asia. Figure S8 shows the location and vertical structure of prescribed heating in the three LBM experiments. In exp. 1, we prescribe the idealized heating in PNWI (red dashed rectangle in Fig. 1A; 67°E-79°E and 24°N-33°N) with climatology during July to August. Keeping the heating configuration in exp. 1, we replace the basic flow with June climatology in exp. 2. Keeping the basic flow in exp. 1, we move diabatic heating to central India (74°E-86°E and 18°N-27°N) in exp. 3, a region with a lot more rainfall in climatology (Fig. 3, B and C). The conclusions remain unchanged if we slightly alter the heating location or change the dataset of basic flow to the National Centers for Environmental Prediction (NCEP) reanalysis in our three experiments.

### Statistical methods

To enable comparisons across a wide range of observations and simulations, all data have been interpolated to a 1° × 1° resolution. Furthermore, we detrend the monthly variables since our study only focuses on interannual variability. A two-tailed Student’s *t* test is applied for the significance test of correlations and composite analysis. The linear regression method is used to explore the influence of SST forcing and internal variability on coupled patterns of Pakistan flooding and East Asian heatwaves in AMIP simulations. An *F* test is used in these linear regression models.
