# Supplementary material for: Dynamic pathway linking Pakistan flooding to East Asian heatwaves
Source: Sci Adv. 2024 Apr 24;10(17):eadk9250. doi: 10.1126/sciadv.adk9250 (PMC11042738; doi:10.1126/sciadv.adk9250)
Supplement: Supplementary file 1 — Figs. S1 to S9 Tables S1 to S3 [file sciadv.adk9250_sm.pdf]

Science Advances
AAAS

Supplementary Materials for

Dynamic pathway linking Pakistan flooding to East Asian heatwaves

Zheng-Hang Fu *et al.*

Corresponding author: Wen Zhou, wen_zhou@fudan.edu.cn; Shang-Ping Xie, sxie@ucsd.edu

Sci. Adv. **10**, eadk9250 (2024)
DOI: 10.1126/sciadv.adk9250

This PDF file includes:

Figs. S1 to S9
Tables S1 to S3

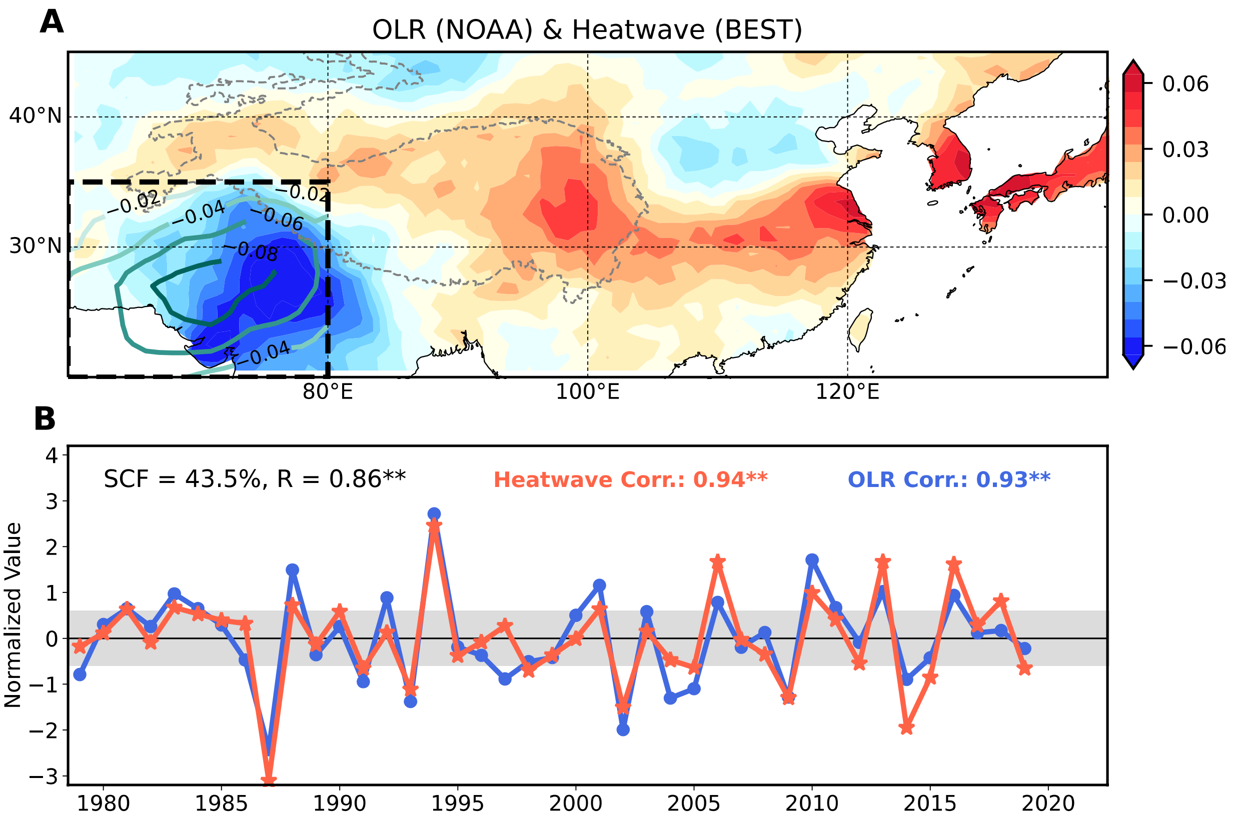

Fig. S1.

Coupled pattern of PNWI flooding and East Asia heatwaves in two other datasets (see Materials and Methods). (**A**) Results for July through August averaged Pakistan–northwest India (PNWI; 60°E–80°E, 20°N–35°N) outgoing longwave radiation (OLR; contours) and East Asia heatwaves (shading) that accompany the first mode of maximum covariance analysis for 1979–2019. (**B**) Time series of the OLR (blue line) and heatwave (red line) patterns, with ±0.6 standard deviation shaded. In (A), gray dashed curves represent the Tibetan Plateau (TP). The squared covariance fraction (SCF) and temporal correlation (R) are denoted at the top of (B). The correlations of temporal coefficients for the OLR (blue lines) and heatwave (red lines) between two suites of datasets are denoted at the top of panel (B). Endings with two asterisks indicate that the correlation is 99% significant in (B).

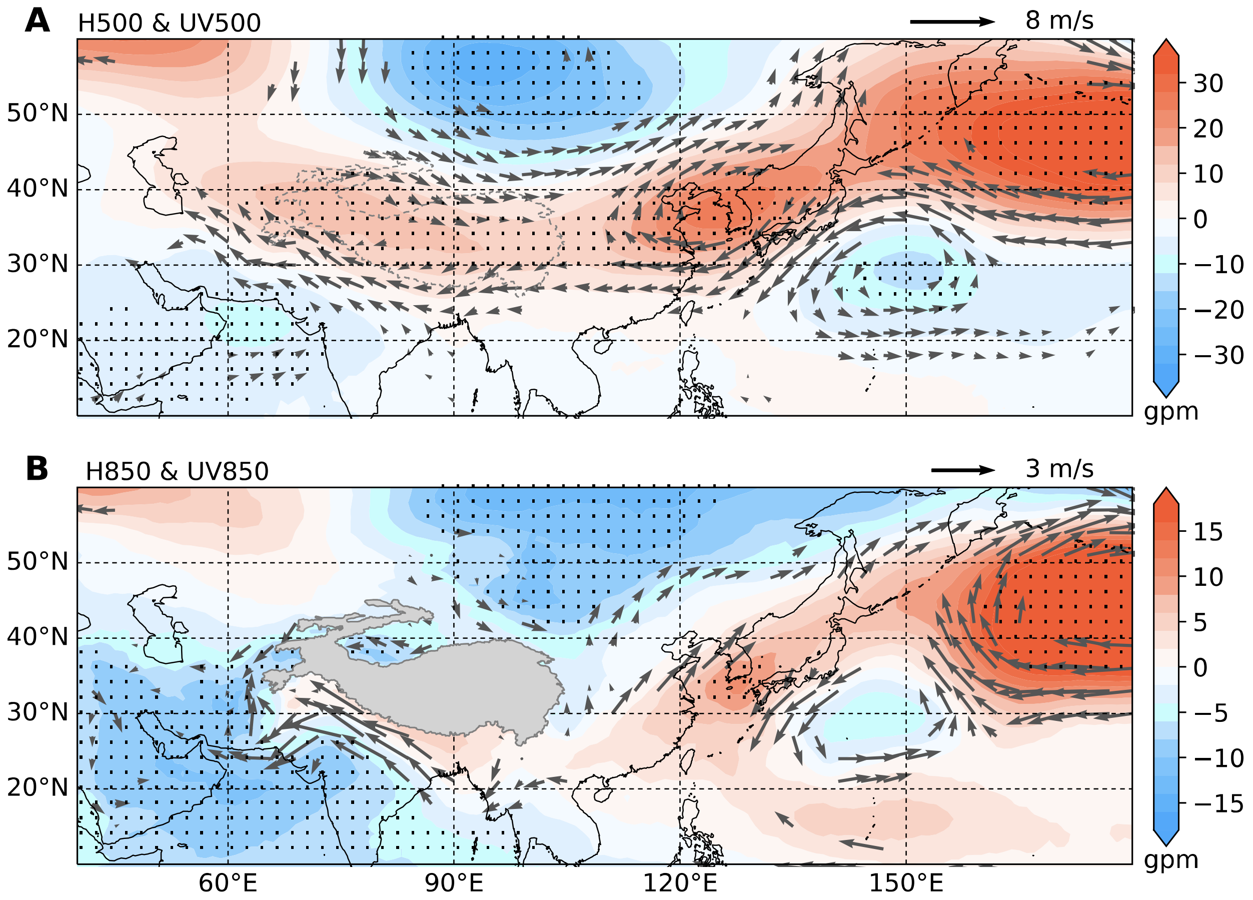

Fig. S2.

Middle and low-level circulation patterns associated with the coupled mode of Pakistan flooding and East Asia heatwaves. Composite pattern differences of horizontal fields for wind (vectors; m s^{-1}) and geopotential (shading; gpm) for (**A**) at 500 hPa, and (**B**) at 850 hPa. The dotted areas and shown vectors indicate that the differences are significant at the 95% confidence level. The TP is denoted by the gray dashed curves in (A) and the gray shaded area in (B).

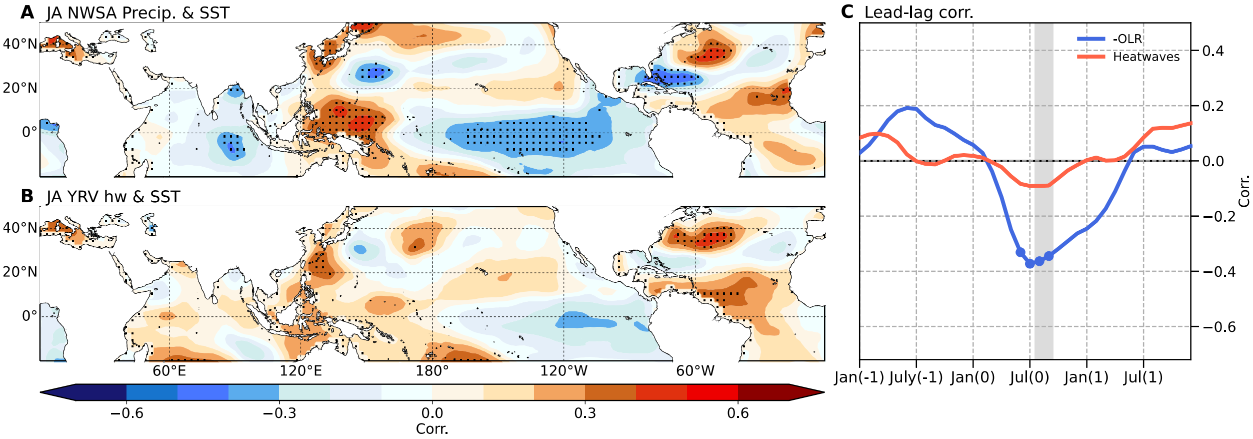

Fig. S3.

Observed relation with the global SST. Correlations between gridded global SST (K) with (**A**) averaged PNWI OLR (multiplied by -1; W m^{-2}), (**B**) averaged Yangtze River valley (YRV) heatwaves (days $year^{-1}$). (**C**) Lagged correlations between the July through August averaged Niño-3.4 index with PNWI OLR (multiplied by -1; blue line) and YRV heatwaves (red line) during 1979–2014. Dots denote that correlations are significant at the 95% confidence level in (A to C). In (C), years -1, 0, and 1 denote the preceding, concurrent, and subsequent years, respectively. Concurrent July to August (JA (0)) is shaded in gray.

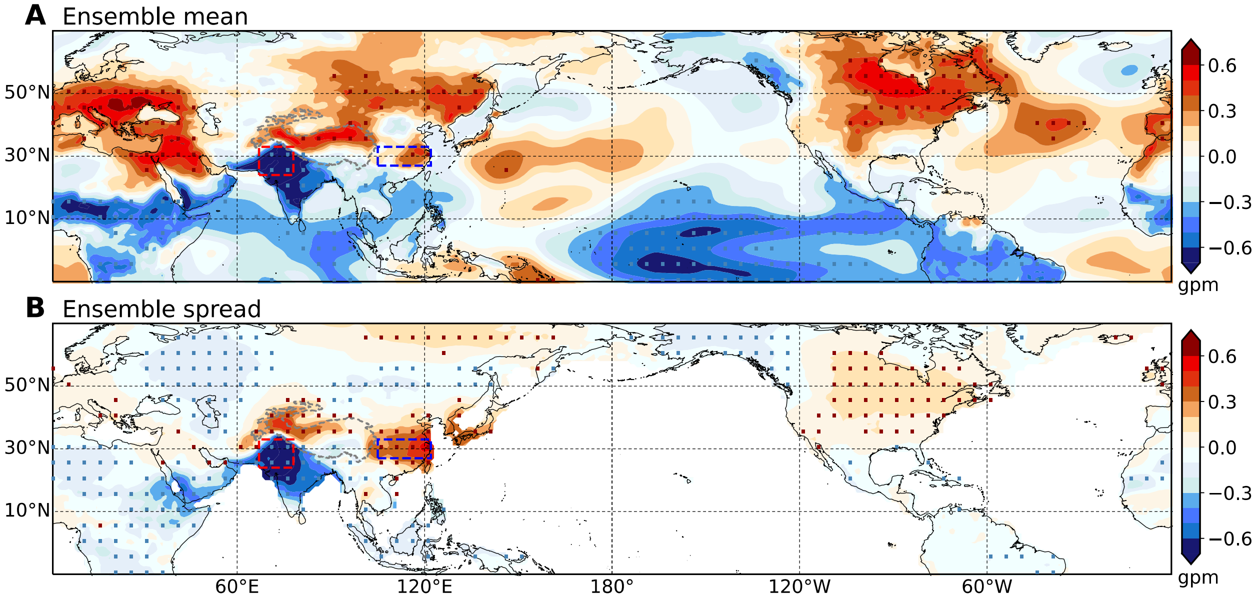

Fig. S4.

Global SST-forced pattern and atmospheric internal pattern in the Northern Hemisphere. Correlations between averaged PNWI OLR (red dashed rectangle; multiplied by -1; W m^{-2}) and gridded surface temperature (K) over East Asia from July through August for the (**A**) ensemble mean, and (**B**) ensemble spread. Dotted regions denote results significant at the 95% confidence level.

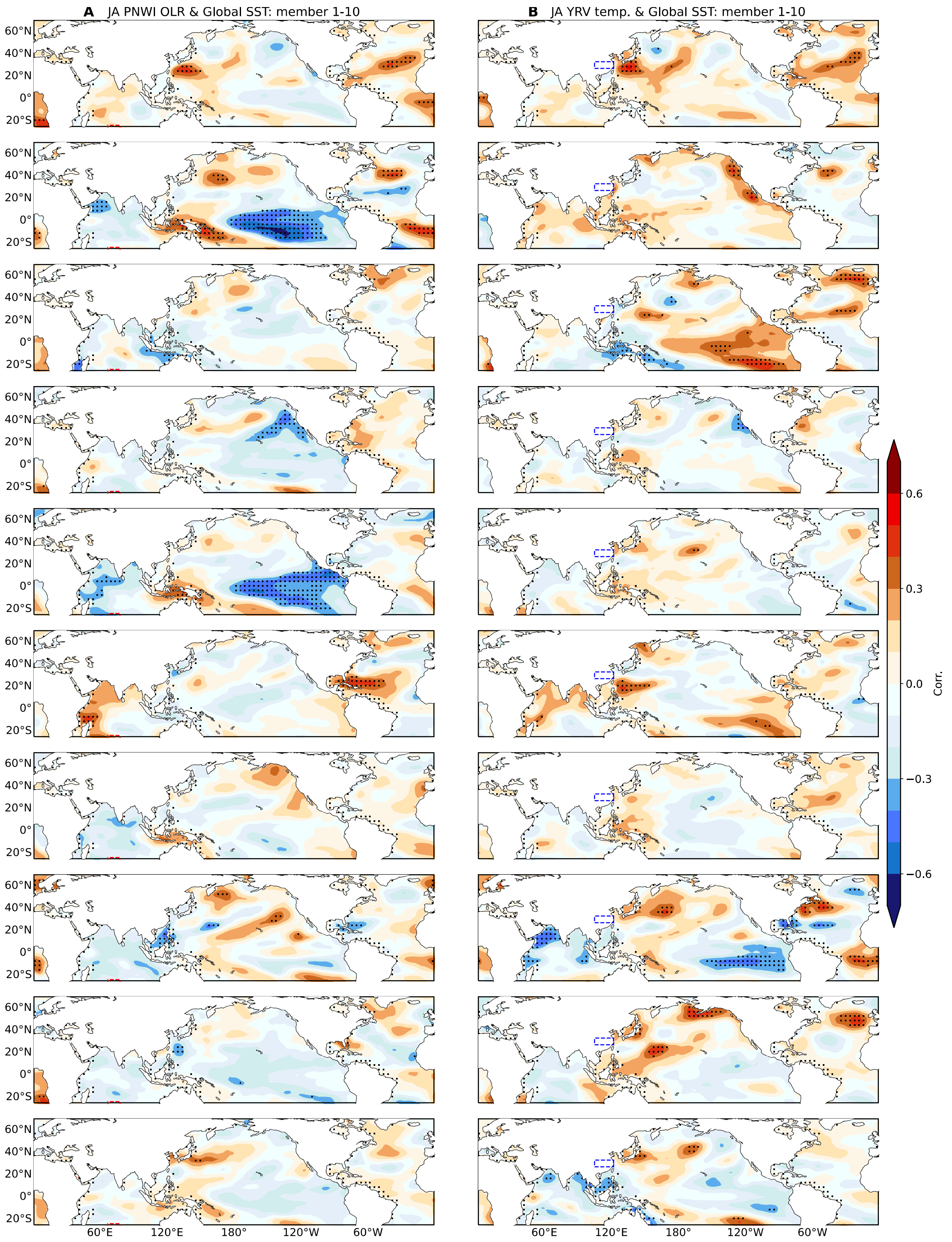

Fig. S5.

Modeled relation with global SST in 10 individual members of AMIP ensemble.
Correlations between the July through August averaged gridded SST with (**A**) PNWI OLR (red dashed rectangle; multiplied by -1; W m^{-2}) and (**B**) YRV surface temperature (K) in the individual members. Dotted regions denote results significant at the 95% confidence level.

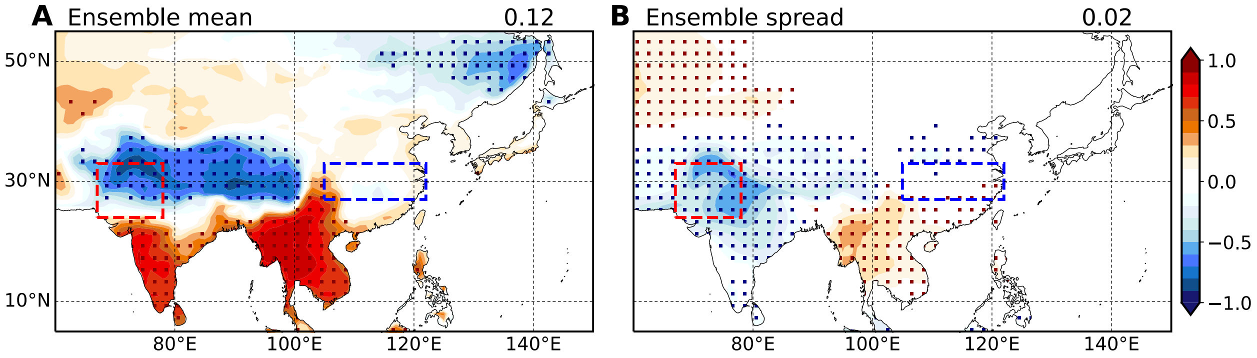

Fig. S6.

Global SST-forced pattern and atmospheric internal pattern in June. Correlations between averaged PNWI OLR (red dashed rectangle; multiplied by -1; W m^{-2}) and gridded surface temperature (K) over East Asia during June for the (**A**) ensemble mean, and (**B**) ensemble spread. The correlations between averaged PNWI OLR and YRV (blue dashed rectangle) surface temperature are denoted in the upper right of panels (A and B). Dotted regions denote results significant at the 95% confidence level.

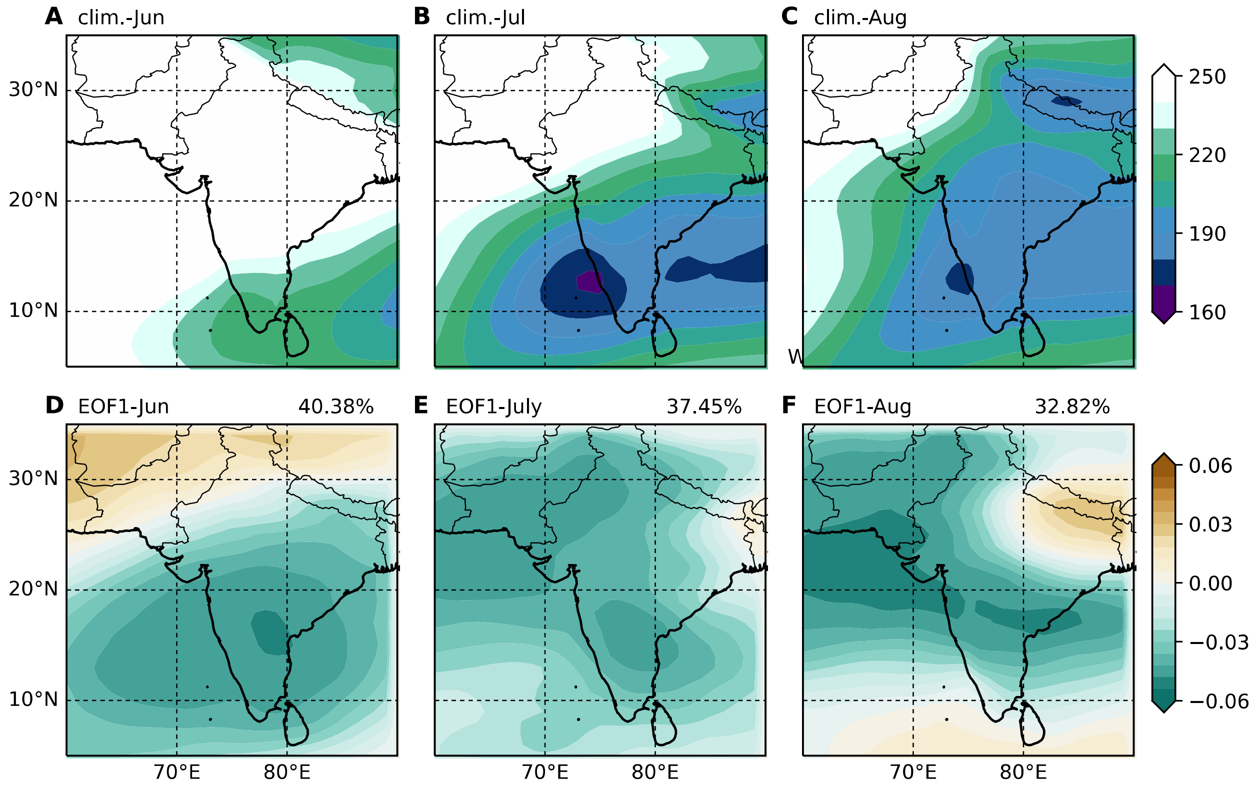

Fig. S7.

Climatology and variability of Indian summer monsoon convection in an atmospheric model run. (**A** to **C**) Monthly mean OLR (shading; W m^{-2}) over the Indian summer monsoon region during 1901–2014. (**D** to **F**) Dominant patterns from monthly empirical orthogonal function (EOF) decomposition for Indian summer monsoon OLR during 1901–2014. Explained variances of the leading modes are denoted at the top of panels (D to F).

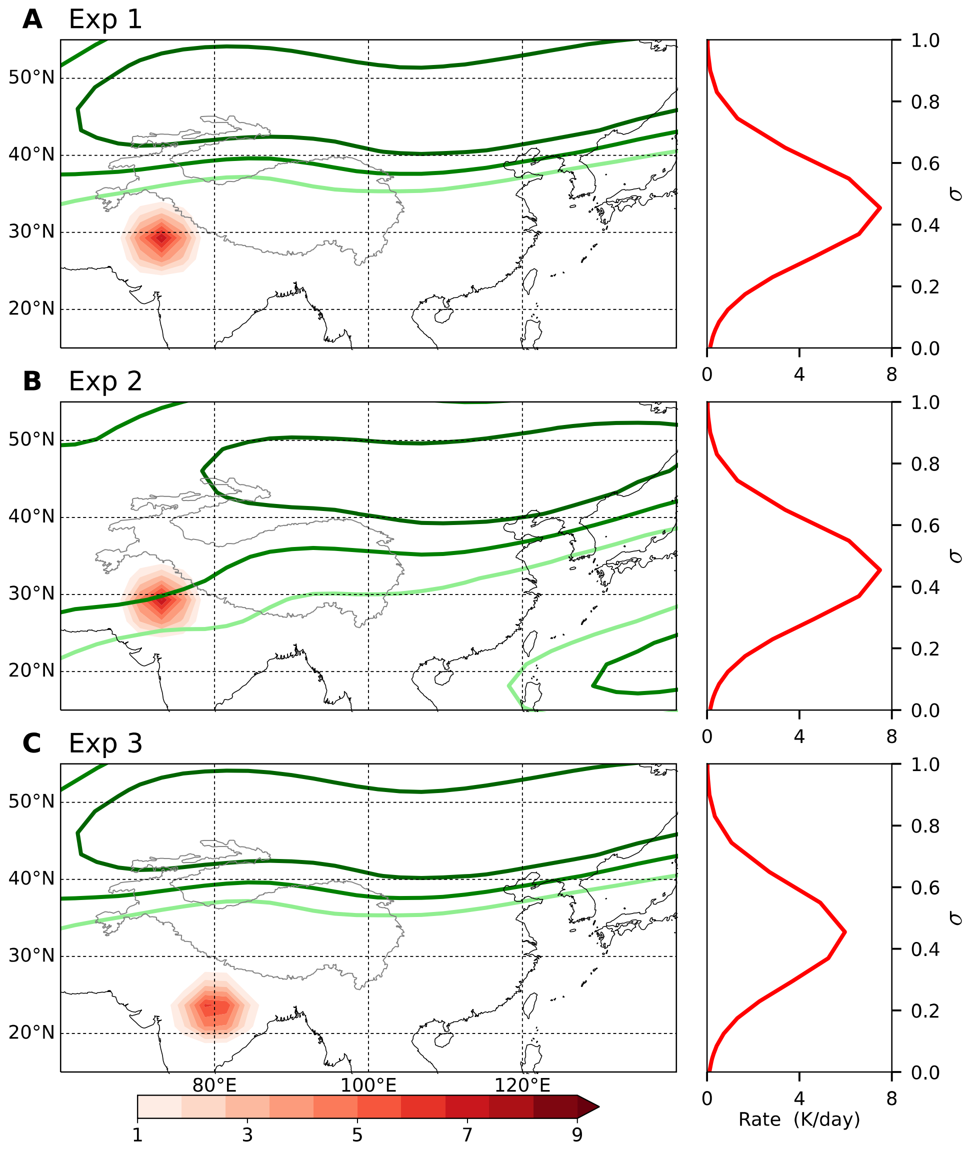

Fig. S8.

Configurations in the three linear baroclinic model experiments. Climatological westerly jet ([10, 15, 20] m/s green contours) and the prescribed diabatic heating (shading; K day^{-1}) at 450 hPa (left panels), and the vertical profiles of the heating rate in the heating center for (**A**) Exp. 1, (**B**) Exp. 2, and (**C**) Exp. 3.

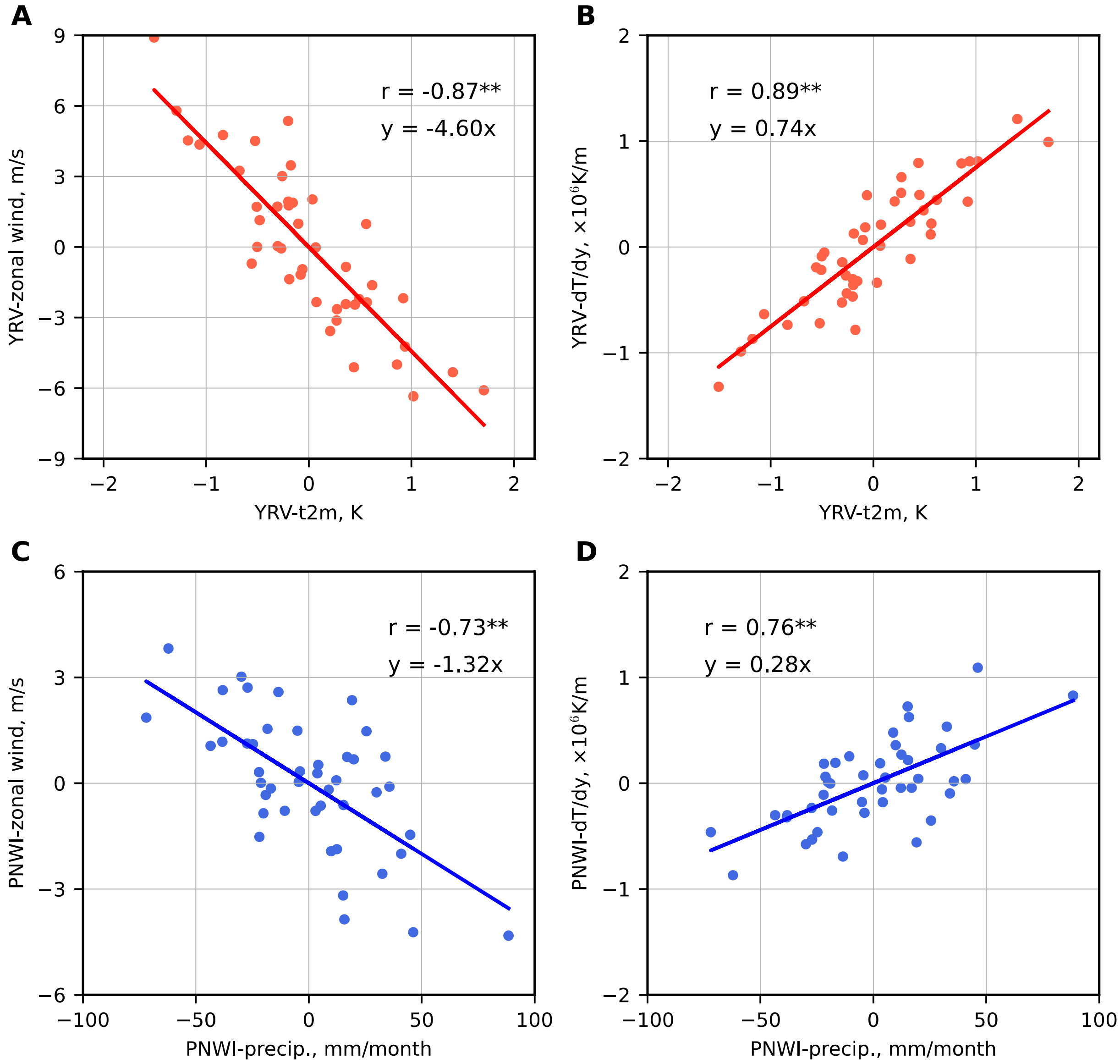

Fig. S9.

Relation between upper-tropospheric zonal wind and surface anomalies. Scatter plots for (**A**) YRV averaged 200 hPa zonal wind anomalies (m s^{-1}) and 2m temperature anomalies (t2m; K); (**B**) YRV averaged meridional temperature gradient anomalies (dT/dy; 10^{-6} K m^{-1}) and t2m (K); (**C**) PNWI averaged 200 hPa zonal wind anomalies (m s^{-1}) and precipitation anomalies (mm $month^{-1}$); and (**D**) PNWI averaged dT/dy (10^{-6} K m^{-1}) and precipitation anomalies (mm $month^{-1}$).

Table S1.
Years included to the positive and negative groups in the composite analysis.

Group	Year
Positive	1983, 1984, 1988, 1994, 2006, 2010, 2011, 2013, 2022
Negative	1987, 1991, 1993, 1999, 2002, 2004, 2005, 2009, 2014, 2021

Table S2.

Observed correlations and partial correlations after removal of the signal of PNWI rainfall between El Niño–Southern Oscillation (ENSO) and YRV heatwaves for 1979–2014. Four precipitation datasets (see Methods) are used for validation. The correlations reaching ±0.33/±0.39 in Table S2 are at a 95%/99% confidence interval.

	YRV heatwaves	ENSO
	ERA5	Berkeley
ENSO	-0.09	0.05
ENSO (no CRU)	0.14	0.26
ENSO (no ERA5)	0.12	0.27
ENSO (no CMAP)	0.08	0.22
ENSO (no GPCP)	0.13	0.25

Table S3.

Relation among PNWI OLR, YRV surface temperature (Temp.), and ENSO in 10 CESM2-CAM6 AMIP runs, in comparison to the observations for 1979–2014. The correlations reaching ±0.33/±0.39 in Table S3 are at a 95%/99% confidence interval.

CESM2-CAM6 member	-OLR & Temp.	-OLR & Temp. (no ENSO)	-OLR & ENSO	Temp. & ENSO	Temp. & ENSO (no -OLR)
1	0.69	0.68	-0.09	-0.08	-0.02
2	0.20	0.21	-0.12	0.07	0.09
3	0.46	0.42	-0.35	-0.22	-0.07
4	0.47	0.50	-0.21	0.10	0.23
5	0.31	0.31	-0.23	-0.07	0.01
6	0.48	0.48	0.00	0.14	0.15
7	0.29	0.30	0.10	0.05	0.07
8	0.52	0.56	-0.19	0.14	0.28
9	0.29	0.30	-0.33	-0.03	0.07
10	0.52	0.51	-0.17	-0.15	-0.07
Observations	0.50 ~ 0.62	0.52 ~ 0.62	-0.35 ~ -0.24	-0.09 ~ 0.05	0.08 ~ 0.27
